# Complete genomic sequences of the single chromosome and four putative plasmids of *Sphingobium yanoikuyae* strain CC4533

**DOI:** 10.1128/mra.01181-25

**Published:** 2026-01-23

**Authors:** Mautusi Mitra, Ana Stanescu

**Affiliations:** 1 University of West Georgia, School of Field Investigations and Experimental Sciences 2291, Carrollton, Georgia, USA; 2 University of West Georgia, School of Computing, Analytics, and Modeling 2291, Carrollton, Georgia, USA; Indiana University, Bloomington, Bloomington, Indiana, USA

**Keywords:** beta carotene-producing, Alphaproteobacterium, xenobiotic degradation, heavy metal tolerance, *Sphingobium yanoikuyae*

## Abstract

A novel *Sphingobium yanoikuyae* strain, CC4533, was isolated from a contaminated tris-acetate-phosphate medium culture plate of a green microalga, *Chlamydomonas reinhardtii*. In this study, we present the complete genome sequence of this beta-carotene-synthesizing, xenobiotic-degrading, and heavy metal-tolerant strain, providing insights into its metabolic capabilities and phylogenetic relationships.

## ANNOUNCEMENT

*Sphingobium yanoikuyae* strain CC4533 was isolated from a contaminated tris-acetate-phosphate medium plate of *Chlamydomonas reinhardtii* wild-type strain CC4533, obtained from the Chlamydomonas Resource Center (https://www.chlamycollection.org/) as part of a local high school student science project at our lab (1). The bacterium was identified as *S. yanoikuyae* (MN633285.1) by partially amplifying and sequencing the 16S rRNA gene (1). Given the strain’s ability to degrade aromatics and hydrocarbons and tolerate toxic concentrations of six heavy metals (1, 2), its genome was sequenced to elucidate the molecular mechanisms underlying these traits.

Genomic DNA was extracted from cells cultured aerobically at 28°C in LB medium using the Qiagen Blood and Cell Culture DNA Mini Kit, following the manufacturer’s protocol without modifications. Sequencing was performed using the PacBio Single Molecule Real-Time (SMRT) platform. DNA shearing was conducted with a Covaris G-TUBE, and fragment size distribution was assessed using a Bioanalyzer 12,000 chip. DNA concentration was quantified using a NanoDrop 2000 spectrophotometer. SMRTbell library preparation was conducted according to the PacBio protocol, targeting 12 kb fragments for purification and concentration. Library quality and barcoding were evaluated prior to sequencing. Barcoded SMRTbell libraries were sequenced on the PacBio Sequel II system using the PacBio sequencing primer v.4 using continuous long read (CLR) sequencing. Adaptor trimming, base correction, assembly, three rounds of polishing, and circularization of reads were performed using the data processing pipeline in SMRT Link v9 software using the HGAP v4.0 pipeline and the Arrow algorithm powered by the PacBio Cromwell workflow engine. The assembly was performed with default parameters, assuming an estimated genome size of 5.5 Mb. For statistical confidence, the genome was also assembled with Flye v2.9.1 (3) and Canu v2.2 (4). Canu v2.2 was used to generate corrected reads. Circlator v1.5.5 (5) was used to detect and trim the overlap to circularize the assembly from the corrected and trimmed reads and the assembly file. Local assembly in Circlator was generated using built-in SPAdes-3.15.4 (6) with a kmer length of 107 bp. *S. yanoikuyae* strain YC-XJ2’s DnaA gene sequence (accession number CP053021.1) was used to rotate the genome using the built-in Pymummer-0.11.0-py3.9.egg in the Circlator. All tools were run with default parameters unless otherwise specified. Statistics of five assembled contigs comprising one circular chromosome and four putative plasmids are in Table 1. PacBio CLR sequencing and assembly statistics, including the QC score, are available at figshare (2).

**TABLE 1:** Assembled contig information^*a*^

Contig and GenBank accession no.	Length (bp)	Circularity	G+C content (%)	Polished assembly coverage (×)
Contig1/chromosome CP115456.1	4,747,868	Yes	65	4,093
Contig 2/plasmid pAS1 CP115458.1	110,920	Yes	60.5	8,463
Contig 3/plasmid pKN1 CP115457.1	647,509	Yes	59.5	3,768
Contig 4/plasmid pMM1 CP115460.1	79,081	Yes	62	6,352
Contig 5/plasmid pTB1 CP115459.1	218,419	Yes	63.5	2,817

^*a*^ Total genome size: 5,803,797 bp.

The assembled genome was deposited in GenBank under accession number GCF_027627615.1. The genome was annotated using the NCBI Prokaryotic Genome Annotation Pipeline (PGAP; v6.10 revision 2025-05-06.build7983; contig gene annotation summary available at https://doi.org/10.6084/m9.figshare.30340213.v3 [2, 7]). NCBI’s CheckM1 genome quality analysis tool (v1.2.3) indicated a completeness of 97.66% and contamination of 14.05% (2, 8), but CheckM2 (a more reliable genome assessment tool; https://github.com/chklovski/CheckM2, release 1.1.0, 20 February 2025 [9]) indicated a completeness of 100% and contamination of 12% (2, 9). The genome was reannotated using RASTtk v1.073 in the DOE Systems Biology Knowledgebase (KBase) with default parameters (10–12). GTDB-Tk v2.3.2 in KBase, with default parameters (12, 13), and PGAP’s built-in ANI tool identified the bacterium as *S. yanoikuyae*. PGAP identified the closest type strain as *S. yanoikuyae* ATCC 51230 (accession number GCA_000315525.1), with an ANI of 96.436% (7, 14), indicating that CC4533 is likely a new strain. Phylogenetic analyses based on the 16S rRNA gene using the KBase Build Phylogenetic Tree (10) from MSA using FastTree2-v2.1.11 application (15) revealed *S. yanoikuyae* strain S72 (accession number CP023741.1) as the nearest neighbor (7, 10).

## Data Availability

The whole-genome sequencing project (including the PacBio DNA methylation motifs) has been deposited at GenBank under the accession number CP115456.1. The raw sequence reads have been deposited in the SRA under the accession number SRR23176675. KBase analysis is publicly available in the KBase static narrative (https://doi.org/10.25982/219679.217/2997510). The PacBio DNA methylome has been submitted to REBASE (Ref#35996).
